# Culturally safe and ethical biomarker and genomic research with Indigenous peoples—a scoping review

**DOI:** 10.1186/s44263-024-00102-0

**Published:** 2024-10-25

**Authors:** Rebecca Dal Pra, Penny O’Brien, Huong X. T. Nguyen, Joanne Luke, Robyn A. Smith, Adrienne Withall, Kylie Radford, Louise M. Lavrencic, Rosie Watson, Leon Flicker, Dina Logiudice

**Affiliations:** 1 Department of Medicine, Faculty of Medicine, Dentistry and Health Sciences, University of Melbourne, Royal Melbourne Hospital, Melbourne, Victoria, Australia, Victoria, Melbourne Australia; 2 Centre for Health Policy, Faculty of Medicine, Dentistry and Health Sciences, University of Melbourne, Melbourne, Victoria, Australia, Victoria, Melbourne Australia; 3 School of Psychology, Faculty of Science, University of New South Wales, New South Wales, Sydney Australia; 4 University of New South Wales Ageing Futures Institute, University of New South Wales, New South Wales, Sydney Australia; 5 Neuroscience Research Australia (NeuRA), New South Wales, Sydney Australia; 6 School of Population Health, Faculty of Medicine and Health, University of New South Wales, New South Wales, Sydney Australia; 7 Department of Population Health and Immunity, Walter and Eliza Hall Institute of Medical Research, Victoria, Melbourne Australia; 8 Royal Perth Hospital, Perth, WA Australia; 9 Western Australian Centre for Health & Ageing, Medical School, University of Western Australia, Perth, WA Australia

**Keywords:** Indigenous health, Genomics, Biomarkers, Culturally safe research

## Abstract

**Background:**

Indigenous peoples globally continue to be underrepresented in biomarker, genomic, and biobanking research. The aim of this study was to identify core components of culturally safe and ethical biomarker and genomic research with Indigenous peoples in Australia, Aotearoa/New Zealand, Canada and the USA.

**Methods:**

A scoping review with a systematic search strategy was conducted utilising electronic databases MEDLINE, EMBASE, PsychINFO, CINAHL and Global Health. Key search terms included ‘biomarkers’ and ‘genomics’ research involving Indigenous peoples in relation to ethical and legal principles of respect, sovereignty, governance and existing policies. Original research studies published from the year 2000 to the 1st of August 2023 were reviewed in a systematic manner. Components of culturally safe and ethical research processes were identified and synthesised descriptively. The quality of included studies was assessed using an Aboriginal and Torres Strait Islander Quality Appraisal Tool through an Indigenous lens.

**Results:**

Seven interrelated research components were identified from seventeen studies as core processes to enhance the cultural safety of biomarker and genomic research. These included building relationships and community engagement, learning, research coordination, logistics, consent, samples and biospecimens, biobank structures and protections and policy. The importance of ensuring self-determination, ownership and decision-making power is emphasised in processes to establish and conduct biomarker and genomic research with Indigenous peoples.

**Conclusions:**

Components that contribute to the cultural safety of biomarker and genomic research processes identified in this scoping review were assembled into a theoretical framework to guide research practice. Further evaluation is required by Indigenous peoples and communities to appropriate and adapt this framework for local use to promote the cultural safety of research processes and minimise barriers to Indigenous peoples’ participation in biomarker and genomic research.

**Supplementary Information:**

The online version contains supplementary material available at 10.1186/s44263-024-00102-0.

## Background

Biomarker and genomic research can involve participants providing radiological data or biospecimens, such as blood or tissue samples [1–4]. Biospecimens, radiological and genetic data may be collected for the purpose of a single research project, or collected and consented for storage in an archive, often termed a biobank or repository, where it can remain for future investigations [5]. The integration of genomic and biomarker technology into healthcare and medical research has expanded clinical knowledge with profound implications on the diagnostic capabilities and potential treatment strategies for many complex diseases [6]. This is widely known in the realm of cancer research where the detection of gene mutations affects cancer subtyping with significant and tangible effects on treatment options, prognostication and outcomes. More recently, biomarkers have become a focus for neurodegenerative diseases such as Alzheimer’s dementia where clinical diagnosis was hitherto the gold standard. The detection of biomarkers such as amyloid, tau and neurofilament light chain can improve early and accurate diagnosis, even in the prodromal stages where cognitive changes have not yet impacted on function. Timely and accurate diagnosis of dementia is vital in the management and support of those affected and their carers [7, 8].

Despite its recent advancement and increasing influence, Indigenous peoples globally are underrepresented in biomarker and genomic research. Barriers to Indigenous peoples’ participation in biomarker and genomic research are influenced by a variety of factors, including researchers’ failure to enact ethical and appropriate community engagement strategies, lack of study transparency, historical and ongoing research misconduct and culturally unsafe research practices, all of which contribute to a reluctance to share personal information, including genetic material, with the research community [9]. In dementia research specifically, biased recruitment processes and selection criteria can also serve to exclude Indigenous peoples and other populations, with significant implications for research findings and their translation into clinical practice [10, 11].

Health and biomedical research have historically not served Indigenous peoples in colonised nations such as Australia (Aboriginal and Torres Strait Islander peoples), Aotearoa/New Zealand (Māori), Canada (First Nations, Inuit and Métis) and the USA (Alaskan Native and American Indian). As Indigenous peoples were systematically colonised, their communities and cultural practices began to be studied, misrepresented and described from the point of view of researchers with more power, privilege and different systems of knowledge [12]. Indigenous peoples have raised concern about the negative impacts and harms associated with past research practices including stigmatisation, violation of individuals’ rights, misuse of samples, reinforcement of ‘victim blaming’ approaches to health inequalities and a lack of benefit for Indigenous peoples and communities [12, 13]. The consequence of this is a legacy of mistrust that impacts Indigenous peoples’ participation in scientific research involving the collection of biospecimens for the study of genes and biomarkers [13].

Over the last decade, efforts to ‘bridge the divide’ and enhance culturally safe research processes have improved the acceptance of genomic research within some Indigenous communities [9]. This is further supported by newly established research centres, guidelines and policies such as that of the National Indigenous Genomic Centre in Australia, the Te Mata Ira Guidelines for Genomic Research with Māori peoples in Aotearoa, and a new culturally informed genetic research policy among the Navajo Nation in the USA that foster new ways to engage with Indigenous communities [14, 15]. The Te Mata Ira Guidelines for Genomic Research with Māori peoples in Aotearoa were developed over the course of a three-year research project that explored diverse Māori views on genomic research and biobanking and resulted in the development of a cultural foundation that provides direction for maintaining cultural authority and authenticity in research processes. Existing guidelines on genomic research also explore ideas on how to conduct culturally safe and ethical research with Indigenous peoples [6]. However, this knowledge is largely conceptual with a lack of practical frameworks, based on primary research and co-design, co-development and data sovereignty principles, available to guide researchers and clinicians in how to conduct biomarker and genomic research that meets the needs, values and preferences of Indigenous peoples. The aim of this scoping review was to identify and synthesise core components of culturally safe and ethical biomarker and genomic research with Indigenous peoples in Australia Aotearoa/New Zealand, Canada and the USA to guide research practice.

## Methods

### Overview and methodological framework

A scoping review was conducted according to the guidance of the Preferred Items For Systematic Reviews and Meta-Analysis extension for Scoping Reviews (PRISMA-ScR) checklist (Additional file 1), to systematically map and synthesise evidence describing components of culturally safe biomarker and genomic research involving Indigenous peoples [16]. No protocol was registered for this scoping review. To ensure clarity in this review, the research question was identified and developed using the Sample, Phenomenon of Interest, Design, Evaluation and Research type (SPIDER) framework [17] (see Table 1) and sought to answer the research question: *What are the components of culturally safe and ethical research processes for conducing biomarker and genomic research with Indigenous peoples?*

**Table 1:** SPIDER [17] elements for scoping review research question

Sample	Indigenous adults residing in Australia (Aboriginal and Torres Strait Islander), New Zealand (Māori), Canada (First Nations, Inuit and Métis) and the United States of America (Alaskan Native and American Indian)
Phenomenon of interest	Indigenous peoples’ participation in cultural and ethical safe genomic and biomarker research
Design	Primary studies with results disclosing Indigenous peoples and researchers’ attitude to culturally safe and ethical research
Evaluation	Identifying the components of genomic and biomarker research or identifying frameworks/policies/guidelines for conducting the genomic and biomarker research
Research type	Qualitative, quantitative or mixed methods

### Search strategy and eligibility criteria

A search strategy relating to medical subject headings (MeSH) and keywords associated with ‘Indigenous peoples’, ‘biomarkers’ and ‘genomics’ was developed with the support of an expert medical librarian and tested and adapted in five electronic medical databases including MEDLINE, EMBASE, PsychINFO, CINAHL, Global Health (see example search strategy in Additional file 2). The search strategy was also originally designed with ‘dementia’ as an additional MeSH and keyword as this scoping review forms part of a larger program of research conducted at the OnTRACK (Teaching Research and Community Knowledges) Centre for Research Excellence (CRE). The OnTRACK CRE is a national program that aims to promote brain health with Aboriginal and Torres Strait Islander peoples. One of the specific aims of OnTRACK is to co-develop a framework for biomarker research to improve dementia diagnosis among Aboriginal and Torres Strait Islander peoples in Australia. Testing the search strategy with 'dementia', resulted in too few search results. The research team decided to broaden the scope of inquiry to include biomarker and genomic research in all health conditions. The authors acknowledge the complexity of using terminology such as biomarker and genomic together or interchangeably and as such include the following definitions. Biomarkers were defined as any molecule in the brain or biological fluids associated with a disease state that facilitates its diagnosis. Considering the role of imaging as a biomarker for dementia, structural and functional neuroimaging markers that are important in diagnosis as a marker of disease states of interest were also included [18, 19]. We define genomics as the study of all of a person’s genes, including the interaction of those genes with each other and the person’s environment. For the purposes of this scoping review, we included both terms as both types of research potentially share similar processes. For instance, biospecimens, genetic samples and genetic data may be collected and stored for use in one or more research projects. Eligibility criteria are presented in Table 2. The search included primary research published after the year 2000. This cut-off date was selected as older publications likely contain out-of-date information. Searches were undertaken on the 1st of August 2023.

**Table 2:** Eligibility criteria

Inclusion criteria	Exclusion criteria
Qualitative, quantitative and mixed-methods studies	Secondary studies, scoping reviews, systematic reviews, conference proceedings, conference abstracts
Studies after 2000	Studies published before 2000
English language	Non-English language
Adults—over 18 years old	Children—under the age of 18 years
Countries listed—Australia, Canada, New Zealand, North America	Indigenous populations from nonnominated countries
Humans, adults	Animal studies, studies in children
Research processes when conducting biomarker and/or genomic research including terms—ethics, consent, permission, authorisation, respect, recognition, dignity, trust, equity or sovereignty, or governance or regulation or arrangement or directions or standards or management or policies or methods or plans or protocol or strategy or guidelines or directive or framework or scheme or biobank or cultural safety or Indigenous data sovereignty	

### Study selection

**Fig. 1:**
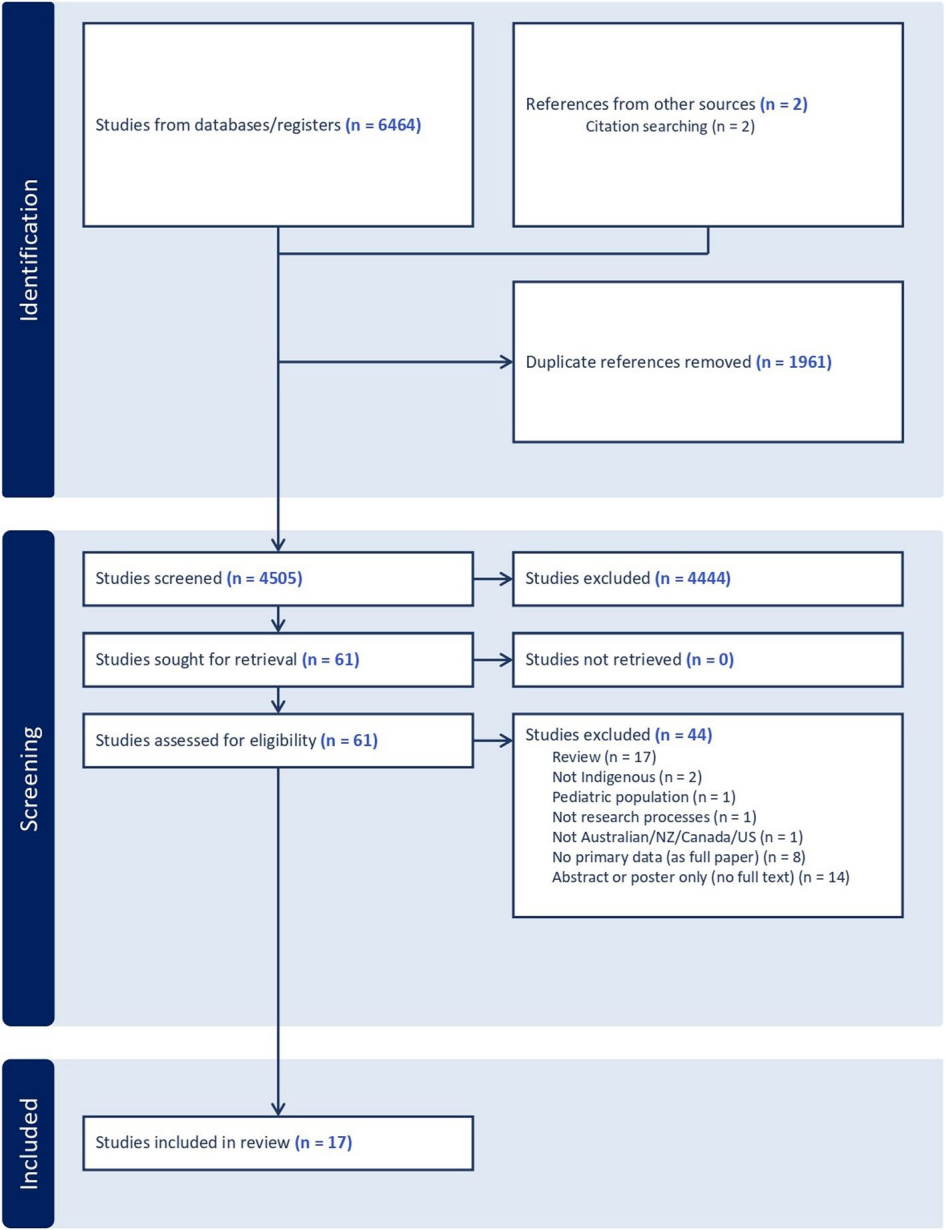
PRISMA flow diagram

Search results were imported into the bibliographic management software Endnote X7 (Clarivate Analytics, PA, USA) to remove duplicate articles. Titles and abstracts were uploaded to Covidence systematic review software (Melbourne, Australia) [20] and screened by two independent reviewers (RP and PO, JZ, HN or JC) and conflicts were resolved through consensus discussions with a third reviewer (DL). Following this, full texts were uploaded and screened using the same process. Finally, reference lists of included full texts were reviewed (citation searching) by members of the research team to identify any additional texts of relevance (Fig. 1).

### Quality assessment

The Aboriginal and Torres Strait Islander Quality Appraisal Tool (QAT), developed by Harfield et al. and adapted for use in an international context, was employed to appraise the quality of the included studies from an Indigenous perspective [21]. An Aboriginal member of the research team (JL) applied the tool, comprising 14 questions to assess the quality of the included studies. The research team was cognizant of the cultural diversity amongst the Indigenous populations included in this study, however agreed that the QAT could be applied here given parallel experiences of colonial research, and shared ethical principles within national and international human rights instruments and ethics statements and guidelines relevant to Indigenous health research [22, 23]. The tool assesses the quality of studies from an Indigenous lens including concepts of Indigenous governance, respect for cultural and intellectual property, capacity building, and beneficial outcomes.

### Data extraction, charting and data synthesis

Data extraction was performed by the first author (RD) using a purpose-designed Microsoft Excel spreadsheet. Extracted data comprised of study characteristics (e.g. title, authors, country of publication, Indigenous population, methodology, study aims, see Additional file 3). From each study, core components of the culturally safe and ethical research process were also identified and synthesised in a descriptive manner. Where appropriate, example quotes from Indigenous participants have been included to privilege Indigenous voices throughout the review.

## Results

### Study characteristics

**Fig. 2:**
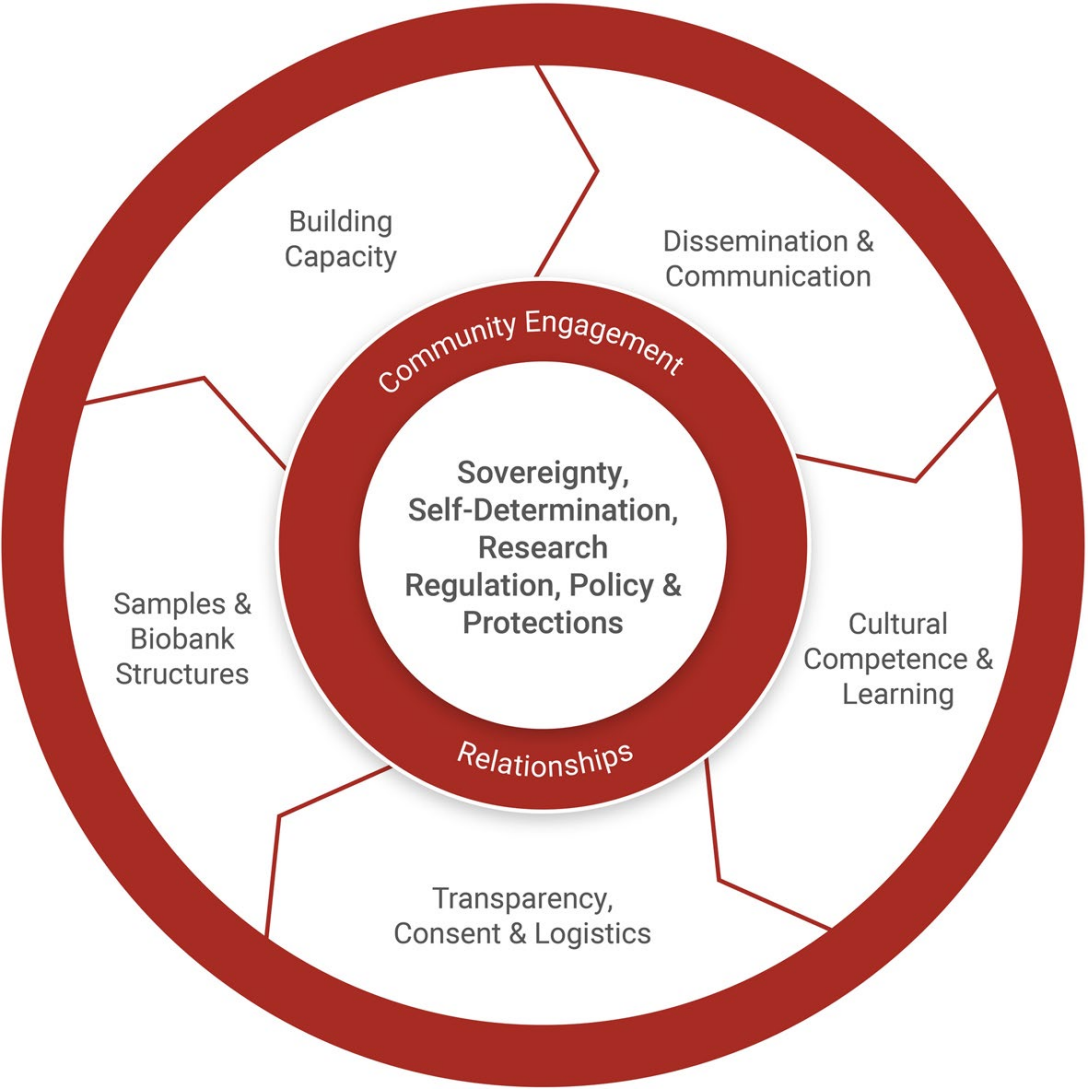
Framework for conducting culturally safe and ethical biomarker and genomic research with Indigenous peoples

The search yielded 6464 studies, of which 1961 were duplicates. A further 4444 studies were excluded after the title and abstract screening, and a further 44 studies were excluded during full-text screening based on the eligibility criteria. Citation searching and expert consultation yielded an additional two full texts. A total of 17 eligible studies published between 2005 and 2023 were included in the final synthesis (see Fig. 2 for flow chart). Of the included studies, most were conducted in the USA (*n* = 9), [24–32] followed by Australia (*n* = 4) [33–36] Canada (*n* = 2) [37, 38] and Aotearoa/New Zealand (*n* = 2) [14, 39]. The majority of the included studies utilised qualitative research methods including interviews [14, 25, 33, 34, 37, 39], focus groups [29, 30, 32, 37, 38] and participatory action research techniques such as workshops, forums and deliberations [14, 24, 26, 28, 35, 36, 39]. One study utilised an expert panel [31] and one study used quantitative methods including a survey [27]. A full summary of the included study characteristics can be found in Additional file 3.

### Quality appraisal using the QAT

Appraisal of the 17 studies using QAT found that most fully or partially included Indigenous leadership, governance and authorship and engaged with a ‘strength-based’ understanding that recognised the harms of colonial research environments, especially in the context of research involving biospecimens. Two main areas of improvement were identified. Firstly, there was an overarching need for research groups to incorporate community-based priority setting, rather than priorities set by universities or non-Indigenous clinicians. Secondly, most studies did not include negotiating agreements regarding rights of access to Intellectual Property (IP) or cultural IP generated from research. Lastly, one study was considered poor quality evidence in terms of its ethical underpinning [27]. The study did not include Indigenous authorship and used outdated, offensive terminology to refer to the included Indigenous population. An overview of the results of this appraisal can be found in Additional file 4.

### Core research components

Language among the 17 included studies regarding components of research varied and was represented in ways such as elements, strengths, remedies, perspectives, obstacles, barriers, and challenges associated with culturally safe and ethical biomarker or genomic research. Seven core components were identified across the studies. A summary of the components that were identified in each study is provided in Table 3.

**Table 3:** Summary of core research components identified in each included study

Components	Relationships and community engagement	Learning	Research coordination and logistics	Consent	Samples and biospecimens	Biobank structures	Protections and policy
Beaton et al. 2017 [39]	✓	✓		✓	✓	✓	✓
Blacksher et al. 2021 [24]	✓	✓					
Caron et al. 2023 [37]	✓	✓	✓	✓	✓		✓
Dalach et al. 2021 [33]	✓	✓	✓				
Donoghue et al. 2021 [35]	✓	✓	✓	✓			
Garrison et al. 2019 [25]						✓	✓
Hermes et al. 2021 [34]	✓			✓	✓	✓	✓
Hiratsuksa et al. 2020 [26]	✓	✓	✓	✓			✓
Howard et al. 2005 [27]	✓	✓	✓				
Hudson et al. 2016 [47]	✓	✓		✓			✓
Johnson et al. 2009 [32]	✓	✓	✓				✓
Kaladharan et al. 2021 [36]	✓	✓	✓	✓			✓
Morgan et al. 2019 [38]	✓			✓		✓	✓
Reedy et al. 2020 [28]	✓	✓		✓		✓	✓
Shaw et al. 2013 [29]		✓	✓	✓			✓
Tauali’i et al. 2014 [30]	✓	✓		✓	✓	✓	✓
Waanders et al. 2023 [31]	✓	✓	✓	✓		✓	✓

### Component 1: Relationships and community engagement

Building relationships grounded in trust and benefit for Indigenous peoples participating in research emerged as the most important contributor to conducting culturally safe and ethical biomarker and genomic research [26, 37]. Such relationships were said to be forged from productive community engagement and discourse that builds legitimate connections between Indigenous peoples and researchers [37, 38]; ‘We speak a lot about community engagement, when you want to go and plan for a community you should go and learn from the community’—First Nations person, Canada [38]. Factoring in the time and appropriate setting for meaningful community engagement throughout all phases of research, such as local community meetings or drop-in centres where potential participants reside, enabled direct discussions and the opportunity for Indigenous peoples and researchers to ask and answer questions [26]. Other participatory methods such as deliberations, forums and workshops co-facilitated by Indigenous community members that allowed time for Indigenous peoples and researchers to interact, set priorities and provide input to research design were also identified as beneficial relationship-building activities. Participants and potential participants reported feeling connected, and respected and that their views on research processes were heard and valued [14, 24, 26, 35, 37]. Dialogue that emerges from authentic relationships and involving Indigenous peoples as collaborative researchers gives further opportunities for Indigenous peoples to make informed decisions about participating in research and guides how data should be used and shared [26, 31, 37].

### Component 2: Learning

Learning was also identified as important in facilitating Indigenous peoples’ participation in biomarker or genomic research [14, 35]. Potential participants and participants should be given local, relevant information on research involvement, research processes and the benefits and risks of biomarker and genomic research [35]. Given the complexity of the topic, any training or education should incorporate appropriate communication and language free from complex medical jargon and be facilitated through trusted sources such as local Indigenous health or medical services. Resources in culturally adapted, accessible formats such as videos that incorporate local languages and artwork were also seen as positive education initiatives to improve genetic health and research literacy [35]. It should be noted that any learning initiatives should not become onerous to participants [33]. In addition to learning aimed at improving genetic health and research literacy, practical and specific cultural safety training was recommended to empower researchers and clinicians to provide greater cultural support, ensure culturally safe research processes and build shared knowledge about Indigenous worldviews to enable effective collaborative relationships [14, 26, 31, 33, 35]. Such cross-cultural learning and cultural safety education may also help to alleviate potential barriers to participation posed by cultural events or traditions [26, 27].

### Component 3: Research coordination and logistics

Some included studies identified that healthcare services or institutions performing research are often physically or financially inaccessible for Indigenous peoples [31, 33]. Geographical distance posed not only a logistical barrier to participation but a cultural barrier, where ‘distant’ researchers lacked community connections and local community knowledge [26, 31]. Efforts should be made by research groups to minimise logistical barriers, including geographical and financial barriers, by providing services such as free transport and ensuring research participation does not impose a cost on communities [31, 33, 38, 40].

### Component 4: Consent

The concept of consent has significant cultural and ethical implications in all research, but especially in the context of biomarker and genomic research. Informed consent should be both community-centred (relating to an Indigenous community’s participation), and person-centred (relating to an individual participant’s enrolment in a study) [14, 36]. Various mechanisms were identified to ensure that consent procedures were meaningful and valid. These include the use of simplified terminology to describe complex genetic, health or research processes and embedding community engagement and education processes (as described above) to ensure that the scope and specificity of the project and guidelines for data sharing, access and use are negotiated and agreed on by the local community and formalised in research agreements [26, 30, 35, 39]. This should also encompass supplementary or secondary use of genetic and biomarker research data held in repositories [26, 37, 39]. Consent procedures may differ significantly between communities and projects, for example, some communities may prefer an active consent arrangement whereby participants must be contacted prior to their data being used in supplementary projects: [26, 30, 34, 37]. ‘For me, one consent form doesn’t mean for everything, you know. I think every time there’s someone coming into the bank with their research, I want them to give me another consent form, you know, for anything and everything.’ [37]. First Nations Person (Canada). In contrast, other participants did not necessarily highlight the need for active and ongoing consent processes, rather valuing detailed information and transparency in initial broad consent procedures: ‘for example, it should spell out in the agreement with their donor [biobank participant] if he or she chooses to keep the sample in the biobank, it should say somewhat what they can do with it—they can say that do whatever you want with it or here’s specific things that you shouldn’t do. Give the authority to my daughter, son or whoever after.’ [37]. First Nations Person (Canada).

### Component 5: Samples and biospecimens

Researchers should be aware and understand the value and connection that some Indigenous peoples have to human tissue and samples, particularly blood, donated for the purposes of biomarker or genomic research [30, 35]. Community consultation about the value attached to biospecimens and by extension, the value of Deoxyribonucleic acid (DNA) and genomic data as a representation of tissue [38, 39]. The contribution of biospecimens including blood should be accompanied by information about how the biospecimens will be used and disposed of post-bequest, as Indigenous people’s connection to their blood and other tissues including fingernails, hair and urine may not end when the research ends [39]. ‘As an Aboriginal person…that blood sample, sacred sample…once its [brought] back, then we might get rid of it. In proper way. Not just chuck it, anywhere in the ground. Well firstly, its very sacred and its got life in it and … to us its very important because in blood there are lots and lots of different ceremonies that are involved.’—Aboriginal man (Australia) [34]. Strategies to enhance Indigenous connection and control over their samples, such as videos that describe the journey that the blood will take following collection, barcode enabling sample tracking via participants’ devices or a destroy option that could be activated in the event of a donor’s death were also suggested [38]. Some participants showed a desire to remain connected to the sample and researchers should be aware of any requirements for the sample to be returned for proper interment or disposal in a culturally sensitive manner [34]. Ultimately, research transparency at each stage is vital to maintain community trust and researcher accountability, especially where samples and biospecimens are involved [14, 30]. ‘There should be a check and balance somehow, and to report back to the people that are part of this gathering and to always have notices out there, somehow, and where it’s accessible.’—Native Hawaiian (USA) [30].

### Component 6: Biobank structures

Biobanks or repositories are collections of human material (including blood, cells, tissues and DNA) that can be used for the purposes of biomedical research, screening or diagnosis. Indigenous peoples had differing views on how to structure biobanks in the most culturally safe and ethical ways. However, the majority of included studies highlighted the importance of Indigenous control at an individual and community level [34, 37–39]. Indigenous peoples may directly govern their data through authorisation measures or have full control with Indigenous-owned and governed biobanks [38]. Maintaining control and access rights over the repositories may help to moderate concerns regarding the misappropriation of participants’ data and the potential to build research capacity within Indigenous communities [38]. Part of maintaining control also included participants' desire to retain veto rights about how their biospecimens and data were used, both at a governance and operational level [39]. Other considerations for ethically establishing or maintaining a biobank with Indigenous participants included exploring the language preferences of participants (e.g. participants becoming members of a biobank, rather than as donors of specimens), considering biobanks with Indigenous-only specimens and considering both the preference of participants to remain anonymous or be identifiable for the purpose of being able to seek further input or consent from participants [25, 34, 37, 39]. Non-sanctioned use of data can be excluded with specific consent clauses [37] or with regular feedback reports to Indigenous peoples who have donated data about the storage of their sample and any proposed further use.

### Component 7: Protections and policy

The ongoing use and proprietorship of the donated samples and data is a critical component [25, 37]. Data should remain in the ultimate control of the donor or where appropriate, control should be delegated to a trusted guardianship or stewardship process. For example, permanent cultural oversight or guardianship of the biospecimens or samples including blood by Indigenous peoples or communities may be an appropriate process to ensure the sanctity of samples or data [39]. Additional miscellaneous protections include the classification of data to exclude any findings that could be linked to Indigenous peoples or their populations, particularly in studies with familial or few participants, unless the individuals or community have provided consent for this [25, 32, 37]. The formalisation or embedding of Indigenous peoples’ authority over their data is consolidated through government policy and guidelines that entrench these powers and rights [14, 37]. This may require advocacy measures or politicization of past failings to create the political will for policy change.

## Discussion

This scoping review identified seven core, interrelated research components that have the ability to enhance the cultural safety of biomarker and genomic research conducted with Indigenous peoples. These included relationships and community engagement, learning, research coordination, logistics, consent, samples and biospecimens, biobank structures and protections and policy.

By its nature, biomarker and genomic research generate a unique set of ethical challenges. Where conducted with Indigenous peoples and within the historical context of Indigenous research, additional considerations must be taken into account to engage Indigenous peoples and communities in ethical ways, to enhance their representation in biomarker and genomic research that may aid in reducing future healthcare disparities [41]. In order to effectively address these challenges, formal guidelines and frameworks are needed to provide greater clarity for researchers, improve ethical and culturally safe research processes and enhance Indigenous peoples’ trust, confidence and control when participating in biomarker and genomic research.

A comparative analysis of Indigenous research guidelines conducted in 2012 also highlighted the need for developing and implementing both community-based and international policies to guide Indigenous leaders, policymakers and researchers in best-practice research processes for conducting genomic research with Indigenous communities [42]. In response to specific historical cases of research misconduct in this field [43–45], and improvements in culturally safe research processes more broadly, some Indigenous communities have begun developing their own guidelines to promote responsible conduct of biomarker and genomic research. Research guidelines such as *Te Mata Ira: Guidelines for Genomic Research with Māori and He Tangata Kei Tua Guidelines for Biobanking with Māori* in Aotearoa and *Guidelines for Genomic Research with Aboriginal and Torres Strait Islander Peoples of Queensland* seek to empower Indigenous peoples to engage and participate genomic research [46–48]. Common recommendations for protecting the rights of Indigenous peoples in biomarker and genomic research across the guidelines include (1) the need to engage, consult and involve the community in the planning of the research as well as throughout the project (including Indigenous governance and leadership), (2) Focusing research on Indigenous health priorities, (3) embedding clear, transparent and ongoing communication process, including feedback of results to community, (4) creating consent processes that meet the needs of the community, (5) embedding capacity building and education into the research and (6) developing local protocols about sample and data collection, storage and use [46, 47]. The *Guidelines for Genomic Research with Aboriginal and Torres Strait Islander Peoples of Queensland* also recommend their guidelines as a starting point for best-practice strategies for Aboriginal and Torres Strait Islander peoples in Queensland, as the policy is specific to genomics and Queensland [46].

More recently, a group of Indigenous scientists and members of the Summer Internship for Indigenous Genomics Consortium and Indigenous community members in the USA have developed the *Framework for Enhancing Ethical Genomic Research with Indigenous Peoples *[9]*.* Despite these steps in the right direction, there remains no international policy on best practice, nor an Australia-wide framework to guide biomarker and genomic research with Aboriginal and Torres Strait Islander peoples. Although the intended aim of this review was to inform the co-development of a framework for investigating biomarkers in dementia diagnosis, insufficient evidence was identified to answer this specific research question. This is in keeping with a recent review by our research team that highlighted a paucity of published biomarker research involving Indigenous peoples worldwide [18].

Broadening our research to an international context to inform a local issue may be a potential limitation of this study. Previous reviews in a similar context have also acknowledged that synthesising data across multiple Indigenous peoples or communities may attempt to homogenise peoples and possibly oversimplify socially and geographically unique cultures [49]. Despite this consideration, many Indigenous peoples across the included studies and the wider literature [9, 50], appear to share similar beliefs, experiences and concerns with genomic research, tissue donations, and biobanking practices. Many of the factors identified in this review also align closely with that of the more recent framework developed by Claw et al. *Framework for Enhancing Ethical Genomic Research with Indigenous Peoples* that arose from active and frequent communication between researchers (including Indigenous researchers) and community members over many years comprised of multiple training workshops, community meetings, and development of digital and print informational materials. The majority of the studies included in this review were also considered high-quality evidence in terms of Indigenous research practices. These frameworks emphasise community engagement and collaboration with local Indigenous communities, the need for cultural competency and education for researchers about local community values and perspectives, education and capacity building for community members to understand and be involved in the research process and the need for transparent, ongoing communication with Indigenous community [9]. This may suggest that the findings from this review are an appropriate foundation to inform the future development of international and local policies to guide culturally safe biomarker and genomic research.

The methodological strengths of this work include the robust search strategy formulated with an expert medical librarian through repeated trialing to ensure that the search outcomes were systematic and comprehensive. However, we acknowledge that this review only captures what has been reported on or published in academic research and potentially misses unpublished knowledge about culturally safe research practices. The seventeen included studies were heterogeneous and demonstrated wide variance in context, research processes and Indigenous peoples involved. It should also be acknowledged that the study screening and data extraction components of this review were undertaken by a non-Indigenous researcher (RD, a Master of Public Health Student) which may have introduced bias in the way the data were synthesised and considered. An Indigenous researcher working alongside RD may have added further insight into the cultural factors associated with research and identified themes. Despite this, the included studies were appraised by an Aboriginal member of the research team, and interpretation of the results and the final manuscript had input from a multidisciplinary team of Aboriginal researchers, clinicians and an Indigenous Reference Group. Further, although the QAT has been used in an international context, it has only been validated for use with Aboriginal and Torres Strait Islander peoples.

Our findings align closely with previously developed ethical frameworks and principles but emphasised the importance of self-determination, ownership and decision-making power in establishing and conducting biomarker and genomic research with Indigenous peoples. The components identified in this review have been used to extend the existing model developed by Claw et al. into a flexible theoretical *‘Framework for conducting Cultural Safe and Ethical Biomarker and Genomic Research with Indigenous Peoples’* that aims to guide research practice (see Fig. 2). We did not include the ethical values of Respect, Reciprocity, Equity and Beneficence included by Claw et al. in our framework as the research team wanted to focus on actionable and practical guidance for researchers. We recommend that this framework is best used as a blueprint or starting point to guide research groups to engage with Indigenous researchers, peoples and communities in shaping their own governing framework to inform study designs and local research policies. For example, research groups seeking to establish a program of genomic research involving Indigenous peoples may input project-specific information into each step of the framework. This will enable the research to be guided and informed by the values of the local community members and be disease-specific where appropriate [4]. Further testing, through the application of the proposed framework to real-world settings in different geographical and social contexts would strengthen its utility.

In addition to extending this framework with the findings of this review, we provide a summary of recommendations for researchers in Table 4. Practical recommendations described here should always be considered alongside the values outlined in local ethical research guidelines, for example, the National Health and Medical Research Council Guidelines for ethical conduct for research involving Aboriginal and Torres Strait Islander People and Communities in Australia [51].

**Table 4:** Summary of practical recommendations

Component identified	Practical recommendations for future research
Relationships and community engagement	• Establish relationships grounded in Indigenous cultural values and perspectives, trust and mutual benefit • Engage and involve the local community in all aspects of the research process from planning to dissemination • Utilise participatory action research methods, engage Indigenous community members as collaborative researchers (capacity building) and establish an Indigenous advisory body • Encourage ongoing dialogue and communication from the pre-research phase to address evolving needs, concerns, and opportunities in the research process
Learning	• Provide jargon-free, learning embedded in strong cultural practices (such as storytelling and yarning sessions for Aboriginal and Torres Strait Islander peoples) to potential participants on research processes, benefits and risks • Co-design and develop resources like videos and brochures in local languages with Indigenous artwork to improve genetic health literacy • Ensure research and project team have been provided in-depth cultural safety training, with a specific focus on cultural elements of genomic and/or biomarker research
Research coordination and logistics	• Understand practical barriers that Indigenous peoples may face in participating in research • Provide free transport or financial assistance to support potential participants • Costs to the communities for research participation must be recognized and reimbursed
Consent	• Design informed consent materials tailored to the needs and preferences of the local community and individuals participating in research
Samples and biospecimens	• Understand and respect the cultural significance of biospecimens by handling (including disposal) them in a way that does not contradict community beliefs and standards • Develop strategies like videos and barcode tracking to enhance community control over their samples • Specimens and knowledge gained from the project ideally should remain under the ownership of the community but under the stewardship of the research team
Biobank structures	• Embed Indigenous governance structures to emphasise Indigenous control of biobanks • Respect participant preferences regarding anonymity and specific/ongoing consent arrangements
Protections and policy	• Establish mechanisms for permanent cultural oversight and guardianship of biospecimens and biobanks by Indigenous peoples • Formalise Indigenous peoples’ authority over their data through government policies • Advocate for policy changes and political support to entrench these powers and right

## Conclusions

This scoping review explored components of culturally safe and ethical, genomic and biomarker research with Indigenous peoples. The seven core components identified as contributing to the cultural safety of biomarker and genomic research processes were assembled into a blueprint theoretical framework to guide research practice. Further evaluation is required by Indigenous peoples and communities to determine the value and utility of this framework to enhance culturally safe research processes and increase Indigenous peoples’ participation in biomarker and genomic research.

## Supplementary Information

Supplementary Material 1.

Supplementary Material 2.

Supplementary Material 3.

Supplementary Material 4.

## Data Availability

All data generated or analysed during this study are included in this published article and its supplementary information files.

## Abbreviations

CINAHL: Cumulated Index in Nursing and Allied Health Literature
DNA: Deoxyribonucleic acid
EMBASE: Excerpta Medical Database
MEDLINE: Medical Literature Analysis and Retrieval System Online
MeSH: Medical Subject Headings
PRISMA-ScR: Preferred Items For Systematic Reviews and Meta-Analysis extension for Scoping Reviews
PsychINFO: Psychological Information Database
QAT: The Aboriginal and Torres Strait Islander Quality Appraisal Tool
USA: United States of America
SPIDER: Sample, Phenomenon of Interest, Design, Evaluation and Research type
