# Supplementary material for: Culturally safe and ethical biomarker and genomic research with Indigenous peoples—a scoping review
Source: BMC Glob Public Health. 2024 Oct 25;2:72. doi: 10.1186/s44263-024-00102-0 (PMC11622903; doi:10.1186/s44263-024-00102-0)
Supplement: Supplementary file 2 — Supplementary Material 2. [file 44263_2024_102_MOESM2_ESM.docx]

**Additional File 2 - Example search strategy**

| ID# | Search terms - Medline | # Studies |
| --- | --- | --- |
| #1 | exp Indigenous Peoples/ | 36095 |
| #2 | exp American Indian or Alaska Native/ or exp Indians, North American/ or exp Navajo People/ or exp Native Hawaiian or Other Pacific Islander/ | 34938 |
| #3 | exp Māori People/ or exp Pacific Island People/ | 12140 |
| #4 | exp Indigenous Canadians/ or exp Inuit/ | 4159 |
| #5 | exp Australian Aboriginal or Torres Strait Islander/ | 149 |
| #6 | (indigenous or indigene* or aborigin* or torres strait islander or torres strait islanders or maori* or polynesian* or pacific peoples or pacific islander or native american or native americans or american indian or american indians or Navajo or amerind* or alaskan* or eskimo* or native hawaiian or native hawaiians or first nation or first nations or first peoples or inuit* or metis or native canadian or native canadians or canadian indian or canadian Indians).mp. | 85206 |
| #7 | exp north america/ or exp canada/ or exp Australia/ or exp New Zealand/ | 1873574 |
| #8 | 6 and 7 | 35887 |
| #9 | ((indigenous or aborigin*) and (australia* or victoria* or new south wales or queensland or tasmania or northern territory)).mp. | 11594 |
| #10 | 1 or 2 or 3 or 4 or 5 or 8 or 9 | 52146 |
| #11 | exp biomarkers/ | 879735 |
| #12 | exp genomics/ |  |
| #13 | ((biological or bio or epigenetic or fluid or genetic or genomic or genome) adj2 marker*).mp. | 93922 |
| #14 | (fluid biomarker or biofluid).mp. | 2110 |
| #15 | (biomarker or biomarkers or genome* or genomic*).mp. | 1576625 |
| #16 | 11 or 12 or 13 or 14 or 15 | 1978175 |
| #17 | 10 and 16 | 2145 |
