# Supplementary material for: Culturally safe and ethical biomarker and genomic research with Indigenous peoples—a scoping review
Source: BMC Glob Public Health. 2024 Oct 25;2:72. doi: 10.1186/s44263-024-00102-0 (PMC11622903; doi:10.1186/s44263-024-00102-0)
Supplement: Supplementary file 3 — Supplementary Material 3. [file 44263_2024_102_MOESM3_ESM.docx]

**Additional File 3 - Study Characteristics**

| **Title** | **Authors** | **Journal** | **Country**  **Study design** | **Population &**  **Sample** | **Data collection & Figures** | **Study purpose** |
| --- | --- | --- | --- | --- | --- | --- |
| **Engaging Māori in biobanking and genomic research: a model for biobanks to guide culturally informed governance, operational, and community engagement activities.** | Beaton, Angela; Hudson, Maui; Milne, Moe; Port, Ramari Viola; Russell, Khyla; Smith, Barry; Toki, Valmaine; Uerata, Lynley; Wilcox, Phillip; Bartholomew, Karen; Wihongi, Helen | Genetics in medicine: official journal of the American College of Medical Genetics 2017;19(3):345-351 | New Zealand  Interviews | Māori | Te Mata Ira Model  Te Ara Tika Guidelines | *“Purpose: He Tangata Kei Tua, a relationship model for biobanks, was developed to facilitate best practice in addressing Māori ethical concerns by guiding culturally informed policy and practice for biobanks in relation to governance, operational, and community engagement activities.”* |
| **Deliberations with American Indian and Alaska Native People about the Ethics of Genomics: An Adapted Model of Deliberation Used with Three Tribal Communities in the United States** | Blacksher, Erika; Hiratsuka, Vanessa Y.; Blanchard, Jessica W.; Lund, Justin R.; Reedy, Justin; Beans, Julie A.; Saunkeah, Bobby; Peercy, Micheal; Byars, Christie; Yracheta, Joseph; Tsosie, Krystal S.; O'Leary, Marcia; Ducheneaux, Guthrie; Spicer, Paul G. | AJOB empirical bioethics 2021;12(3):164-178. United States 2021 | North America  Deliberation. | American Indian  Alaka Natives |  | *“In question was how to design deliberations for tribal communities and whether this adapted model would achieve key deliberative goals and be well received.”* |
| **Partnering with First Nations in Northern British Columbia Canada to Reduce Inequity in Access to Genomic Research** | Caron, Nadine R.; Adam, Wilf; Anderson, Kate; Boswell, Brooke T.; Chongo, Meck; Deineko, Viktor; Dick, Alexanne; Hall, Shannon E.; Hatcher, Jessica T.; Howard, Patricia; Hunt, Megan; Linn, Kevin; O'Neill, Ashling | International Journal of Environmental Research and Public Health 2023;20(10): Switzerland 2023 | Canada  Focus Groups  Interviews | Metis  Inuit  First Nations  Northern BB Initiative (NBI) – 6 phase research projects. |  | *Project’s Aim:*  *“(1) increasing awareness of the NBI, biobanking, and genomic research among First Nations community members; and (2) identifying First Nations Peoples’ understanding and experience of biobanking, perceptions of key issues, challenges associated with biobanking, and recommendations or expectations for establishing governance for the NBCFNB in a beneficial and culturally safe way”* |
| **"This is my boy's health! Talk straight to me!" perspectives on accessible and culturally safe care among Aboriginal and Torres Strait Islander patients of clinical genetics services.** | Dalach, Philippa; Savarirayan, Ravi; Baynam, Gareth; McGaughran, Julie; Kowal, Emma; Massey, Libby; Jenkins, Misty; Paradies, Yin; Kelaher, Margaret | International Journal for Equity in Health 2021;20(1):1-13. BioMed Central 2021 | Australia  Interviews | Aboriginal and Torres Strait Islander people | National Health Genomics Policy Framework 2018-2021 | *“This is the first study to explore barriers to accessing clinical genetics services among Aboriginal and Torres Strait Islander people, which has been acknowledged as a key strategic priority in Australian genomic health policy.”* |
| **Referral pathways: Integrated genetic healthcare for aboriginal and Torres Strait Islander Queenslanders** | Donoghue, V.; Pratt, G.; Dingli, K.; Houghton, K.; Young, A.; McGaughran, J. | **Twin Research and Human Genetics 2021;24(5):331. Netherlands Cambridge University Press 2021** | Australia  QLD  Workshops  Consultation methodology. | Aboriginal and Torres Strait Islander people | Genetic Health QLD. | *“Integrated Genetic Health Care: Improving Access for Aboriginal and Torres Strait Islander People to Clinical Genetics through Partnership and Primary Health Leadership, we aim to describe culturally safe and appropriate integrated healthcare and referral pathways to support and improve access for Aboriginal and Torres Strait Islander peoples to clinical genetic services.”* |
| **Access and Management: Indigenous Perspectives on Genomic Data Sharing** | Garrison, Nanibaa' A.; Barton, Krysta S.; Porter, Kathryn M.; Mai, Thyvu; Burke, Wylie; Carroll, Stephanie Russo | Ethnicity & Disease 2019;29():659-668. Owings Mills, Maryland Ethnicity & Disease, Inc. 2019 | North America  Interviews | American Indians  Alaska Natives  Native Hawaiians | NIH Genomic Data Sharing (GDS) Policy | *“This study aims to better understand the specific concerns held by AI/AN/NH leaders and researchers who are engaged in tribal, regional, and national discussions about genetic research as the basis for informing collaborative approaches to data management.”* |
| **Beyond platitudes: a qualitative study of Australian Aboriginal people's perspectives on biobanking** | Hermes, Azure; Wiersma, Miriam; Kerridge, Ian; Easteal, Simon; Light, Edwina; Dive, Lisa; Lipworth, Wendy | Internal Medicine Journal 2021;51(9):1426-1432. Malden, Massachusetts Wiley-Blackwell 2021 | Australia | Aboriginal  people | National Centre for Indigenous Genomics (NCIG) Biobank | *“To explore the perspectives of Australian Aboriginal people whose tissue – or that of their family members – has been stored in the biobank of the National Centre for Indigenous Genomics (NCIG).”* |
| **Alaska Native genomic research: perspectives from Alaska Native leaders, federal staff, and biomedical researchers** | Hiratsuka, Vanessa Y.; Hahn, Michael J.; Woodbury, R. Brian; Hull, Sara Chandros; Wilson, David R.; Bonham, Vence L.; Dillard, Denise A.; Avey, Jaedon P.; Beckel-Mitchener, Andrea C.; Blome, Juliana; Claw, Katrina; Ferucci, Elizabeth D.; Gachupin, Francine C.; Ghazarian, Armen; Hindorff, Lucia; Jooma, Sonya; Trinidad, Susan B.; Troyer, Jennifer; Walajahi, Hina; Avey Jp, Beckel-Mitchener A. C. Blome J. Claw K. Ferucci E. D. Gachupin F. C. Ghazarian A. Hindorff L. Jooma S. Trinidad S. B. Troyer J. Walajahi H.; Alaska Native Genomics Research Workshop, Group | Genetics in medicine: official journal of the American College of Medical Genetics 2020;22(12):1935-1943. United States 2020 | North America  Workshops | Alaska Natives | Alaska Area Specimen Bank (AASB)  AASB policy & procedure | *“The goal of the workshop was to better understand barriers to and opportunities for engaging AN people in genomic research and explore the circumstances and policies that facilitate partnership and participation of AN peoples in community-driven genomics research.”* |
| **A genetic and epidemiologic study of cardiovascular disease in Alaska natives (GOCADAN): design and methods** | Howard, B. V.; Devereux, R. B.; Cole, S. A.; Davidson, M.; Dyke, B.; Ebbesson, S. O.; Epstein, S. E.; Robinson, D. R.; Jarvis, B.; Kaufman, D. J.; Laston, S.; MacCluer, J. W.; Okin, P. M.; Roman, M. J.; Romenesko, T.; Ruotolo, G.; Swenson, M.; Wenger, C. R.; Williams-Blangero, S.; Zhu, J.; Saccheus, C.; Fabsitz, R. R.; Robbins, D. C. | International Journal of Circumpolar Health 2005;64(3):206-221. Finland 2005 | North America  Alaska  Demographic information, physical examination, and survey | Alaska natives | GOCADAN Study | “*In this article we describe the objectives, design and methods of the Genetics of Coronary Artery Disease in Alaska Natives (GOCADAN) study, which was be undertaken to elucidate genetic and environmental factors contributing to CVD.”* |
| **Key informant views on biobanking and genomic research with Māori.** | Hudson, Maui; Southey, Kim; Uerata, Lynley; Beaton, Angela; Milne, Moe; Russell, Khyla; Smith, Barry; Wilcox, Phillip; Toki, Valmaine; Cheung, Melanie | The New Zealand medical journal 2016;129(1447):29-42 New Zealand 2016 | New Zealand  Interviews  Workshops | Māori | Te Mata Ira Project  Te Ara Tika—Guidelines on Māori Research Ethics | *“The aim of the Te Mata Ira project was to explore Māori views on biobanking and genomic research, and to identify ways to address Māori concerns over the collection and use of human tissue.”* |
| **Decisions To Participate in Fragile X And Other Genomics-Related Research: Native American and African American** | Johnson, Vanessa A.; Edwards, Karethy A.; Sherman, Stephanie L.; Stephens, Lancer D.; Williams, Wendy; Adair, Alonna; Deer-Smith, Mary Helen | Journal of Cultural Diversity Fall2009 2009;16(3):127-135 Lisle, Illinois Tucker Publications, Inc. 2009 Fall2009 | North America  Focus Groups. | Native Americans |  | *“Specific aims of the study were to describe, compare, and contrast among and between Native-American and African-American adults as to the following: 1) factors that encourage or discourage decisions to participate in genomics-related research, 2) beliefs about the influence of health care networks accessed to facilitate or discourage participation in genomics-related research, 3) recruitment preferences to encourage participation in genomics-related research, 4) the association between the type of health problem and the likelihood of participation in genomics-related research”* |
| **Ask the people: developing guidelines for genomic research with Aboriginal and Torres Strait Islander peoples**. | Kaladharan, S.; Vidgen, M. E.; Pearson, J. V.; Donoghue, V. K.; Whiteman, D. C.; Waddell, N.; Pratt, G. | BMJ Global Health 2021;6(11):UK BMJ Publishing Group 2021 | Australia  Workshops.  Forums. | Aboriginal and Torres Strait Islander peoples | Genomic Partnerships: Guidelines for Genomic Research with Aboriginal and Torres Strait Islander people s of Queensland. | *“The project aimed to determine preferred practice for genomics research involving Aboriginal and Torres Strait Islander participants based on consultation.”* |
| **Indigenous peoples and genomics: Starting a conversation.** | Morgan, Jenny; Coe, Rachel R.; Lesueur, Rochelle; Kenny, Ruth; Price, Roberta; Makela, Nancy; Birch, Patricia H.; Abadie, R. Heaney K. Alper J. S. Beckwith J. Amendola L. M. Robinson J. O. Hart R. Biswas S. Lee K. Bernhardt B. A. Blout C. Arbour L. Cook D. Bardill J. Garrison N. A. Bate P. Robert G. Beaton A. Hudson M. Milne M. Port R. V. Russell K. Smith B. Wihongi H. City of Vancouver Claw K. G. Anderson M. Z. Begay R. L. Tsosie K. S. Fox K. Garrison N. A. Cochran P. A. L. Marshall C. A. Garcia-Downing C. Kendall E. Cook D. Mccubbin L. Cornel M. C. Bonham V. L. Dodson M. Williamson R. First Nations Health Authority Hudson M. Southey K. Uerata L. Beaton A. Milne M. Russell K. Jacobs B. Roffenbender J. Collmann J. Cherry K. Lee Bitsoi L. M. Bassett K. Evans C. H. Karczewski K. Francioli L. Lek M. Karczewski K. J. Minikel E. V. Samocha K. E. Banks E. Fennell T. Li J. Z. Absher D. M. Tang H. Southwick A. M. Casto A. M. Barsh G. S. Li J. Z. Absher D. M. Tang H. Southwick A. M. Casto A. M. Ramachandran S. Myers R. M. Lionel A. C. Costain G. Monfared N. Walker S. Reuter M. S. Hosseini S. M. Marshall C. R. Manrai A. K. Funke B. H. Rehm H. L. Olesen M. S. Maron B. A. Szolovits P. Kohane I. S. Mathew S. S. Barwell J. Khan N. Lynch E. Parker M. Qureshi N. McElfish P. A. Narcisse M. R. Long C. R. Ayers B. L. Hawley N. L. Aitaoto N. Kadlubar S. McKenzie H. A. Varcoe C. Brown A. J. Day L. McWhirter R. Nicol D. Savulescu J. Pope C. Ziebland S. Mays N. Popejoy A. B. Fullerton S. M. Pullman D. Arbour L. J. O. Young C. G. Brunk Q. S. R. Smith L. T. Statistics Canada TallBear Tan T. Y. Dillon O. J. Stark Z. Schofield D. Alam K. Shrestha R. White S. M. Tarailo-Graovac M. Shyr C. Ross C. J. Horvath G. A. Salvarinova R. Ye X. C. van Karnebeek C. D. Truth; Reconcilliation Commission of Canada, Warren N. S. Wilson P. L. | Journal of Genetic Counselling 2019;28(2):407-418  United Kingdom Wiley-Blackwell Publishing Ltd. United Kingdom 2019 | Canada  Focus Groups | Metis  Inuit  First Nations | Truth and Reconciliation Commission of Canada’s (2015 | *“We therefore aimed to begin a conversation with Indigenous Canadians living in British Columbia, Canada, regarding the consequences (both negative and positive) of their non‐representation in genomic databases.”* |
| **Deliberations About Genomic Research and Biobanks with Citizens of the Chickasaw Nation** | Reedy, J.; Blanchard, J. W.; Lund, J.; Spicer, P. G.; Byars, C.; Peercy, M.; Saunkeah, B.; Blacksher, E. | Frontiers in Genetics 2020;11():466 | North America.  Forums | American Indian – Chickasaw Nation members | Center for the Ethics of Indigenous Genomic Research (CEIGR) consortium National Institutes of Health Center of Excellence in ELSI Research (CEER)  Fig. 1 Deliberation process | “Consortium members and the Chickasaw Nation Department of Health Administration designed a deliberative forum for Chickasaw citizens to consider the potential benefits and risks of participating in genomic research and biobanks.” |
| **Risk, Reward, and the Double-Edged Sword: Perspectives on Pharmacogenetic Research and Clinical Testing Among Alaska Native People** | Shaw, JL., PhD, Renee Robinson, PharmD, MPH, Helene Starks, PhD, MPH, Wylie Burke, MD, PhD, and Denise A. Dillard, PhD | **American Journal of Public Health. December 2013, Vol 103, No. 12.** | North America  Focus Groups | Alaska  Natives |  | *“We aimed to understand the key concerns and priorities of the AN people regarding the use of pharmacogenetics in Alaska’s tribal health care system.”* |
| **Native Hawaiian views on biobanking** | Tauali I, Maile; Davis, Elise Leimomi; Braun, Kathryn L.; Tsark, JoAnn Umilani; Brown, Ngiare; Hudson, Maui; Burke, Wylie | Journal of Cancer Education 2014;29(3):570-57, <Blank> Springer Nature 2014 | North America  Focus Groups | Native Hawaiians | ‘Imi Hale Native Hawaiian Cancer Network | *“This study explored Native Hawaiian perceptions of and expectations for biobanking.”* |
| **Indigenous peoples and inclusion in clinical and genomic research: Understanding the history and navigating contemporary engagement.** | Waanders, Angela; Brown, Alex; Caron, Nadine R.; Plisiewicz, Alexa; McHugh, Sean T.; Nguyen, Thinh Q.; Lehmann, Kaitlin; Stevens, Jeffrey; Storm, Phillip J.; Resnick, Adam; Davidson, Tom Belle; Mueller, Sabine; Kline, Cassie | Neoplasia (New York, N.Y.) 2023;37():100879  United States 2023 | North America.  Canada.  Moderated expert panel. | American Indian – Mohegan Tribe.  First Nation – Sadamok Anishnawbek.  Aboriginal Man. |  | *“Aim to better understand Indigenous Peoples’ perspective on participation in clinical research. The group also endeavours to participate in initiatives aimed at inclusion of Indigenous Peoples in research decisions and program development and to inspire members of their organizations to adopt strategies to reach and benefit Indigenous populations.”* |
